# Electro-mechanical Lung Simulator Using Polymer and Organic Human Lung Equivalents for Realistic Breathing Simulation

**DOI:** 10.1038/s41598-019-56176-6

**Published:** 2019-12-24

**Authors:** Richard Pasteka, Mathias Forjan, Stefan Sauermann, Andreas Drauschke

**Affiliations:** 1 University of Applied Sciences Technikum Wien, Department of Life Science Engineering, Vienna, 1200 Austria; 2 Brno University of Technology, Department of Biomedical Engineering, Brno, 61600 Czech Republic

**Keywords:** Techniques and instrumentation, Electrical and electronic engineering, Medical research, Biomedical engineering, Fluid dynamics

## Abstract

Simulation models in respiratory research are increasingly used for medical product development and testing, especially because *in-vivo* models are coupled with a high degree of complexity and ethical concerns. This work introduces a respiratory simulation system, which is bridging the gap between the complex, real anatomical environment and the safe, cost-effective simulation methods. The presented electro-mechanical lung simulator, xPULM, combines *in*-*silico*, *ex-vivo* and mechanical respiratory approaches by realistically replicating an actively breathing human lung. The reproducibility of sinusoidal breathing simulations with xPULM was verified for selected breathing frequencies (10–18 bpm) and tidal volumes (400–600 ml) physiologically occurring during human breathing at rest. Human lung anatomy was modelled using latex bags and primed porcine lungs. High reproducibility of flow and pressure characteristics was shown by evaluating breathing cycles (n_Total_ = 3273) with highest standard deviation |3σ| for both, simplified lung equivalents (${{\boldsymbol{\mu }}}_{\dot{{\bf{V}}}}$ = 23.98 ± 1.04 l/min, μ_P_ = −0.78 ± 0.63 hPa) and primed porcine lungs (${{\boldsymbol{\mu }}}_{\dot{{\bf{V}}}}$ = 18.87 ± 2.49 l/min, μ_P_ = −21.13 ± 1.47 hPa). The adaptability of the breathing simulation parameters, coupled with the use of porcine lungs salvaged from a slaughterhouse process, represents an advancement towards anatomically and physiologically realistic modelling of human respiration.

## Introduction

Animal testing helped catalyse rapid advancement in many areas of medical engineering by providing insights into the complex functionality of living organisms. Nevertheless, ethical and economic concerns have been raised for years^1^. Because of these concerns, the principle of 3Rs (Replacement, Reduction and Refinement) must be followed before including animals into any testing procedure^2,3^. General consensus dictates to avoid using animal tests whenever reliable and consistent alternatives are available^4^. These efforts have been instigated also by legislative powers e.g. the EU Directive 2010/63/EU^5^ and EU regulation 2019/1010/EU^6^, which have been formed with the aim of reducing the number of animals used for testing. Hence development and validation of new alternatives to animal testing are encouraged^7^.

In medicine, simulation techniques are adopted mainly for equipment testing, treatment planning and medical staff education. Simulation devices comply with the increasing requirements on patient safety and provide educational opportunities in a standardised manner. The field-specific applications of simulation devices are also growing^8^. One of the most important driving forces for the development of models in respiratory research is the increasing incidence of pulmonary disease amongst the world’s population. Deeper insight into the respiratory process would increase development potentials for respiratory care devices and treatment techniques. The latter is particularly important with a growing portion of the population suffering from obstructive and restrictive pulmonary diseases^9^. The Organisation for Economic Cooperation and Development (OECD) has reported, that 6.1% of the population in Europe aged 15 years or older suffers from asthma. The chronic obstructive pulmonary disease (COPD) further affects 4.0% of the same population group according to the EU wide health survey^10^. The ability of a person to breathe is negatively influenced by both asthma and COPD, thus, significantly affecting the quality of life. The modelling of the affected organs in the human body is a state of the art method to extend the knowledge through specific tests.

Depending on the experimental measurement setup the respiratory models can be divided into *in-vivo* models, *in-vitro* testing procedures, *in*-*silico* models and mechanical lung simulators. The first category covers *in-vivo* measurement setups employing laboratory animals or human probands during testing. The complex systemic response of the organism to the particular event which is nearly impossible to simulate otherwise can be obtained using such techniques^11^. According to the 3R principle^2,3^,*in-vivo* studies should be limited and replaced by alternative approaches whenever applicable.

Representing the second category of *in-vitro* techniques is the emerging field of microfluid based lab-on-chip technology. This technology commonly focuses on specific layers of cell cultures and their various interactions on a cellular level. Simultaneously, up to four cell types may be included. Lung-on-chip technology provides valuable insides about the toxicity of tested substances and in virology studies^12^. The third category of *in*-*silico* models currently in use is based on the mathematical simulation of general transport equations^13^. Adjustments of the *in*-*silico* models according to individual airway geometries and adaptability of initial and boundary conditions can provide information about the physical airflow field in sub-millimetre areas^14,15^. The fourth category of mechanical based respiratory models, in general, simulate human breathing process by volume displacement within defined compartments. The compartments used vary depending on the focused problematic and can include electro-mechanically driven syringes, bellows systems or pneumatically driven cylinder systems. The volume displacement can be driven by an external device, most commonly a medical ventilator, or by simulator’s active components e.g. servo-motors^16–18^. The mechanical models can in general accurately control simulation parameters (tidal volume, respiratory rate, etc.) making a simulation of the breathing patterns physiologically authentic. Additionally, they can offer an inexpensive alternative to *in-vivo* animal testing approaches, due to the wide range of usable components^19^. More sophisticated mechanical models are able to represent anatomical structures of the human respiratory system via the inclusion of realistic lung equivalents and simulate further physiologically occurring respiratory processes such as pleural depression. Moreover, various pathological conditions such as airway obstructions can be flexibly simulated by introducing resistor components. Experimental measurements with mechanical simulators are used as a basis for validating numerical models of the respiratory system, medical product development and aerosol inhalation studies. Additional application fields include teaching and practical training of students and medical staff for the use of medical ventilators^20–23^.

Further optimisations of mechanical simulators should consider the respiratory physiology for simulation and modelling purposes. The basic respiratory flow parameters which shall be considered, depending on the accuracy of the simulation, are the breathing frequency (*f*), tidal volume (*V*_T_), inspiratory flow rate (${\dot{V}}_{{\rm{INS}}}$), expiratory flow rate (${\dot{V}}_{EXP}$), inhalation time (*T*_INS_), exhalation time (*T*_EXP_) and breath-holding time (*T*_HOLD_)^24,25^. Taking these parameters into account, the respiratory airflow can be characterised as a periodic, time-dependent function of an incompressible, viscous media^25^.

In this work, the electro-mechanical lung simulator xPULM is used. The underlying concepts and functional evidence with previous generations of the lung simulator have been described by David *et al*. and Forjan *et al*.^26,27^. Based on these results xPULM as used in this work, was developed. The bellow system described in^27^ was again used as a driving mechanism of the volume displacement during the simulation replacing a piston-cylinder system^26^. The xPULM additionally includes changes of the operating system, control software and hardware. In previous generations, as described in^26^, the computational power of the integrated PC influenced the chosen breathing parameters like frequency and tidal volume. In order to meet the highly demanding and complex boundary conditions of a realistic breathing simulation, a real-time acquisition and processing unit was now implemented as a major change. This development stage allowed to further optimize the already well-established methodology of mechanical breathing modelling. The simulation options, therefore, include measurements with different lung equivalents, adjustable tidal volumes and breathing frequencies. The xPULM control software of this version is implemented on a real-time data processing unit making the simulation parameters independent of the computational power of the connected PC. For legal reasons, it was necessary to rename the new model to xPULM. This paper aims to introduce the electro-mechanical lung simulator xPULM in first applications in respiratory research, teaching and training. Design, development and final realisation of the xPULM, closely following the 3R principles, are described. The functionality of the simulator is demonstrated by reliable and reproducible simulation of breathing patterns with various respiratory simulation parameters. The main distinguishing feature of xPULM, in comparison to other respiratory models, is the capability of measuring breathing characteristics with the inclusion of simple or complex replicas of human lungs. Modelling of respiratory simulation processes is thereby moved closer to anatomically realistic lung simulation. Potential application, impact and deployment of the simulator in e.g. testing, teaching and research applications are outlined in the discussion. A video clip, recorded during measurements with primed porcine lungs, highlighting the simulation process is uploaded as a part of the submission.

## Mathematical Model of the Simulator

**Figure 1:**
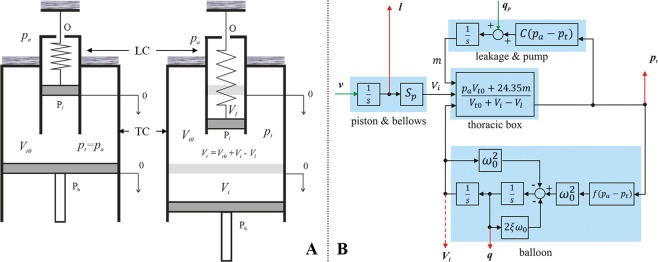
The mathematical model of (**A**) an electro-mechanical simulator with a thoracic chamber *TC* and a lung cylinder *LC* as a simulation basis including the observation parameters of volume *V* and pressure *p* inside *TC* and *LC*. (**B**) The simulation scheme of the simulator including the modelled components of ‘piston & bellows’ (with the cross-sectional area *S*_*p*_), the ‘thoracic box’ (with the atmospheric pressure *p*_*a*_), the volume of the thoracic *V*_*t*_, the initial volume of the chamber *V*_*t*0_, its added volume *V*_*i*_, the corresponding lung volume *V*_*l*_ and - assuming the ideal gas equation - the function of a leakage flow *m*, a model for ‘leakage & pump’ (with leakage constant *C* depending on the pressure difference between atmospheric pressure *p*_*a*_ and pressure in the thoracic chamber *p*_*t*_) and the behaviour of the lung equivalent as a ‘balloon’ (with its dynamic properties characterised by eigenfrequency *ω*_0_ and damping coefficient *ξ*) (based on and adapted from^28^).

The electro-mechanical simulator is based on a mathematical model of the entire system, as shown in Fig. 1. The governing equations were first derived by Solc *et al*.^28^. The basis of the mathematical model is a simulation of the thoracic chamber and a lung equivalent both approximated by pneumatic cylinders in Fig. 1A. The volume of the thoracic chamber can be expressed as:

1 $${V}_{t}={V}_{t0}+{V}_{i}-{V}_{l}$$

where *V*_*t*_ [dm^3^] is the volume of the thoracic chamber, *V*_*t*0_ [dm^3^] is the initial volume of the thoracic chamber, *V*_*i*_ [dm^3^] is the volume added by the movement of the piston *P*_*b*_ and *V*_*l*_ [dm^3^] is the volume of the lung equivalent. The position of the piston is controlled by its velocity. Therefore, the volume added to the thoracic box by the movement of the piston *P*_*b*_ depends on the velocity of the piston *v* [dm/s] and the cross-sectional area of the piston *S*_*p*_ [dm^2^]:

2 $$\frac{\partial {V}_{i}}{\partial t}={S}_{p}v$$

The ideal gas law can be utilised to state the relation between *V*_*t*_ and the pressure inside the thoracic chamber *p*_*t*_ [kPa]:

3 $${p}_{t}{V}_{t}=nRT$$

where *n* [mol] is the amount of gas, *R* = 8.31 J mol^−1^ K^−1^ is the universal gas constant and *T* = 293.15 K is the absolute temperature. The final formula expressing the relationship between volume and pressure inside the thoracic chamber is derived based on the following assumptions: (I) Pressure in the thoracic chamber and in the lung equivalent is equal to the atmospheric pressure at the beginning of the breathing cycle ${p}_{t}\cdot {V}_{t}$ = ${p}_{a}\cdot {V}_{t0}$, (II) the thoracic chamber cannot be considered perfectly airtight and the leakage flow is represented by the function *m*, and (III) the temperature of the air is presumed to be constant 20 °C. Taking into account the listed assumptions, converting pressure units to [bar] and using Eqs. (3) and (1) yields:

4 $${p}_{t}=\frac{{p}_{a}{V}_{t0}+24.35m}{{V}_{t0}+{V}_{i}-{V}_{l}}$$

The time rate of leakage is expressed as:

5 $$\frac{\partial m}{\partial t}=C({p}_{a}-{p}_{t})=C{p}_{v}$$

where *C* [mol s^−1^ bar^−1^] is the leakage constant and *p*_*v*_ [bar] is the vacuum in the thoracic box. To compensate for the leakage, a vacuum pump *q*_*p*_ was included as a further component into the lung simulator. The last step in deriving the complete mathematical model of the lung simulator is to describe the behaviour of an artificial lung equivalent (balloon). Taking the already introduced lung cylinder as the basic modelled of a lung equivalent, the relation between the volume of the lung equivalent *V*_*l*_ [dm^3^], the compliance of the lung equivalent *C*_*l*_ [dm^3^/bar] the damping coefficient *ξ*, the eigenfrequency *ω*_0_ [rad/sec] and the vacuum in the thoracic chamber *p*_*v*_ [bar] is:

6 $$\frac{{\partial }^{2}{V}_{l}}{{\partial }^{2}t}+2\xi {\omega }_{0}\frac{\partial {V}_{l}}{\partial t}+{\omega }_{0}^{2}{V}_{l}={\omega }_{0}^{2}{C}_{l}({p}_{v})$$

where both the damping coefficient *ξ* and the eigenfrequency *ω*_0_ characterise the dynamic behaviour of the elastic balloon.

The initially abstract mathematical description of the model and the underlying processes of volume and pressure changes during respiration were captured and simulated as an interaction of electric and mechanical components. Test measurements have shown^28^ good correspondence between the mathematical model and mechanically realised simulator for both, square and sinusoidal input signals. The shape of the sinusoidal waveform essentially correlates with the simplified flow pattern of human spontaneous breathing at rest^29^. The fundamental theoretical assumptions could thus be confirmed and served as a basis for the development of the current version of the simulator.

## The Electro-Mechanical Lung Simulator - xPULM

**Figure 2:**
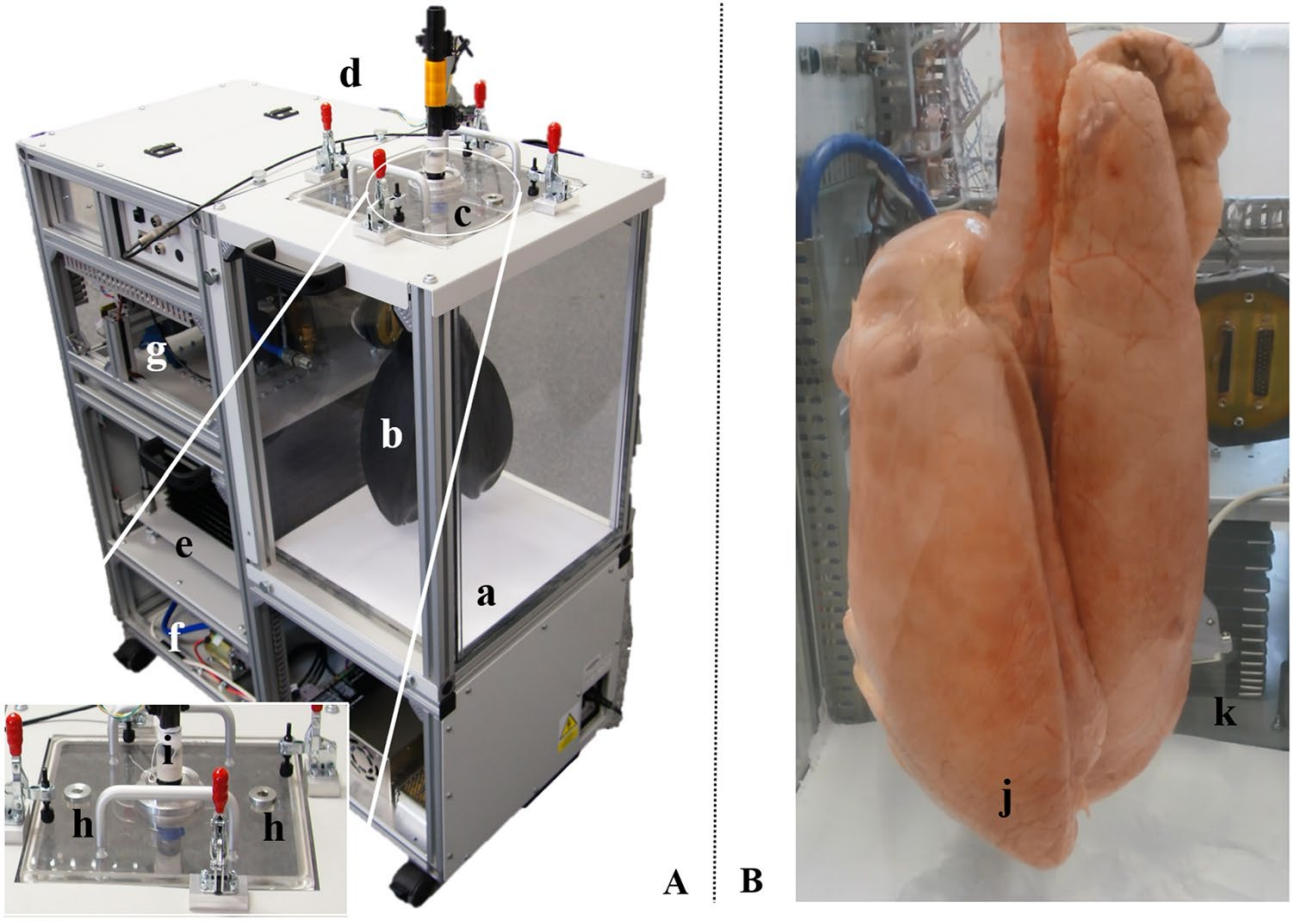
xPULM simulator (**A**) with (a) the thoracic chamber, (b) latex bags as lung equivalents, (c) interoperable lid, (d) airflow sensors (e) the respiratory drive system – realised as bellows system for pressure change generation in the thoracic chamber, (f) the motor with the vacuum pump and (g) the real-time FPGA based control unit. The zoomed-in picture of the interoperable lid shows (h) the connector ports for the organ nutrition circuit (arterial and venous connection) and (i) the distal end of the simulated trachea, used for connecting the aerosol measurement system. Thoracic chamber (**B**) housing (j) a primed porcine lung ventilated with the movement of the (k) bellow system.

This chapter introduces the physical construction, sensory equipment and components interconnections of the electro-mechanical lung simulator, xPULM. The simulator has been developed to replicate physiologically realistic human respiration cycles. The entire setup of xPULM, with main components highlighted, is shown in Fig. 2. The main functionalities include the capability of performing a breathing simulation with changing flow profiles of breathing patterns (sinusoidal), adaptability of simulation parameters (frequency and tidal volume), and the exchangeability of lung equivalents (artificial or organic-based). The mechanical simulator is designed to allow both active and passive respiratory simulation. The term active means that the lung model can be operated to simulate a spontaneously breathing human lung. In contrast, the term passive refers to the ability of the lung simulator to act as a mechanically ventilated human lung, for example using medical ventilators, regardless of their configuration.

**Figure 3:**
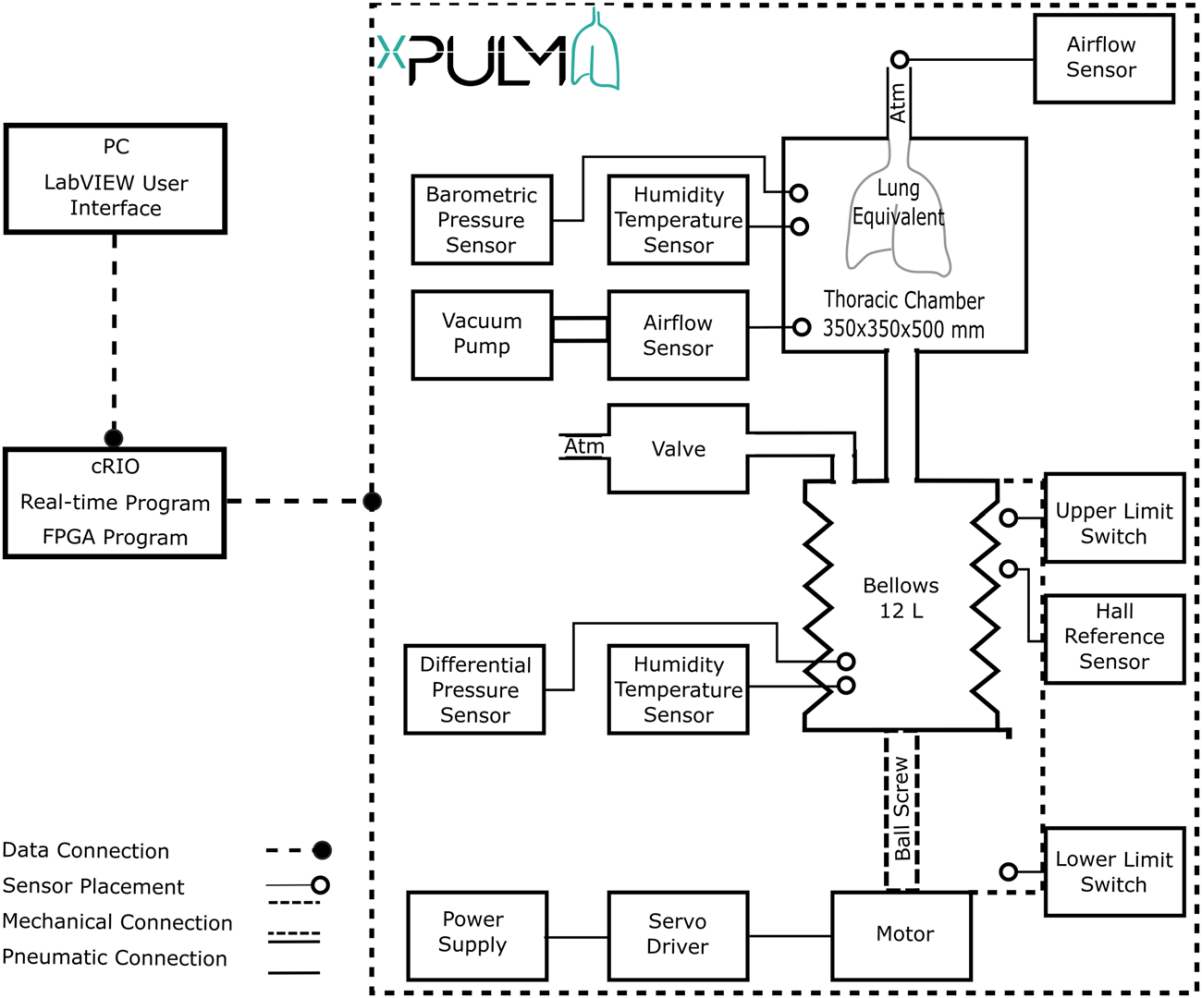
Overview of the xPULM simulator components. This schematic includes interconnection and placement of the simulator main components and sensory equipment for flow, pressure and temperature measurements. All electronic components are connected to the cRIO data acquisition unit. Readings from the sensors are processed by LabVIEW based xPULM control software (Real-time and FPGA program). Simulation parameters can be adjusted using the User Interface running on a dedicated PC.

The construction of the simulator has been inspired by the functionality of the human respiratory system under physiological conditions. The key concept for the inflow and outflow of air into the lungs is the result of the pressure gradient between the atmosphere and the thoracic cavity that was integrated into the lung simulator. Therefore, analogies can be found between the main components of the simulator and functional elements of the human respiratory system. An overview of the simulator setup, depicted in Fig. 3, consists of a respiratory-drive system, the rigid thoracic chamber and an aluminium housing frame. In this construction, the thoracic chamber is the central part of the simulator setup. It houses the chosen lung equivalent and mimics the pressure condition of the thorax in the human body. Moreover, the sensors for monitoring pressure, temperature and humidity changes during simulation are included there. The thoracic chamber is produced out of a transparent Polymethylmethacrylate (PMMA) hosting a total volume of 61 l. This construction allows the simulation process and lung equivalent status monitoring. This feature is particularly useful in training and education.

The lung equivalent is connected to the removable square shaped lid of the thoracic chamber via a threaded connection. Interchangeability of lung equivalents, representing the human lower respiratory tract, is one of the essential elements for anatomically realistic breathing simulation with xPULM. The thoracic chamber can house any chosen lung equivalent constricted only by the maximum volume of the chamber. The structurally simplest equivalent used is an elastic bag (made of latex or silicone), which may vary in volume, form and elastic properties. Depending on the intended simulation, either a single bag may be used or - to achieve a higher degree of approximation to human physiological values - two bags connected via a y-shaped piece may be included. Symmetrical as well as asymmetrical setups of the lung equivalent are possible. As such, any medically relevant lung volume and capacity can be simulated including typical values from small children to adults. For an anatomically more realistic simulation of the human lung, specifically focusing on the internal structure of the lung tissue, a porcine lung may be introduced into the setup instead of polymer-based bags. Thereby the complex inner structures and branching of the respiratory tract from bronchi to the terminal bronchiole are present. This includes bends, bifurcations, and series of cartilage ring supporting the trachea from the inside. Additionally, high humidity levels (≈99% RH), normal in human lungs, comparable lung capacity (3–6 l) and division into lung lobes are represented by using the porcine lungs. Fine structures within the alveolar duct may be occluded due to the preservative process which leads to tissue adhesion especially in the lowest regions of the respiratory tract. Gas exchange is not retained when using a chemically preserved porcine lung. From the construction point of view, the thoracic chamber is connected to the bellows system and the vacuum pump via a separate flange. The airflow created by the vacuum pump is measured by the unidirectional airflow sensor AWM5104VN (Honeywell, USA). The vacuum pump has been introduced into the setup of the xPULM system to achieve similar conditions to the anatomical environment of the human lung, where the organ is attached to the thoracic cage via the pleural liquid and thereby stretched to an extended position constantly. The alterations of the airflow during breathing pattern simulation with xPULM is not caused by the vacuum pump operation. On the contrary, the task of the vacuum pump is to maintain negative pressure inside the thoracic chamber, to counter leakages and to ensure that the inflexion points of the lung tissue are reached throughout the simulation cycle. Subsequently, the incorporated pressure control keeps the lung equivalent from completely collapsing at any point in time during the breathing simulation. The vacuum pump evacuates the thoracic chamber with a peak flow of 5 l/min reaching the maximal negative pressure of −30 hPa (in relation to the atmospheric pressure). To protect the lung equivalent and bellows system from damage the magnetic coil valve is opened in case of excessive negative pressure (*p*_*t*_ < −25 hPa). This leads to pressure equalisation between the atmosphere and the thoracic chamber. The range of negative pressure values during simulations depends heavily on the used lung equivalent and can be set as a parameter in the xPULM control software. The simulation of respiratory patterns with the xPULM is achieved by the movement of the bellows system mimicking exertion of the diaphragm and intercostal muscles during the respiratory cycle. The lower end of the respiratory drive system is movable while the upper part is stationary, fixed to the housing frame of the simulator. Mechanically, the respiratory drive system is realised by a ball screw connected to the movable bottom part of the bellows on the one side and on the other side with a motor via a threaded connection, Fig. 3. A brushless AC servomotor turns the ball screw and the rotatory motion is subsequently transferred to a vertical movement of the bellows system. The range of motion of the entire respiratory drive system is 23.5 cm generating a maximal peak flow of ±130 l/min. Thus, it is guaranteed that any normally occurring physiological or pathological respiratory flow patterns can be simulated. The bellows are protected against potentially damaging over-extension by two mechanical switches. The switches are secured at the simulator housing frame and toggled whenever the upper or lower limit position is reached. Movement can be performed only away from the activated limit position switch. Additionally, the bellow system range of movement is limited in the control software. Furthermore, the signal from a hall sensor serves as a reference point and is used to calculate the initial bellow position during the simulation initialisation procedure. Similarly to the movement of the diaphragm in a human body, motion of the bottom part of the bellows downwards causes the inflation of the lung while the return to the initial position leads to the deflation of the lung. Consequently, the volume displacement in the thoracic chamber manifests as inhalation (extension) and exhalation (compression) of the lung. Physiologically occurring intrapulmonary pressure gradient is accordingly present in the model and leads to the airflow in- and out of the lung equivalent. The in-line airflow through the simulated trachea is measured, using two high precision, unidirectional, temperature compensated airflow sensors AWM720P1 (Honeywell, USA) which are mounted on top of the simulated trachea.

To be able to process the data received from the sensors and to dynamically react on condition changes in real-time, the xPULM simulator is equipped with the embedded industrial data processing and acquisition unit cRIO (National Instruments, USA). This unit consists of a real-time controller 9024 running the xPULM real-time program and a reconfigurable FPGA (Field Programmable Gate Array) chassis 9114 which is flashed with the xPULM FPGA program. The chassis includes I/O modules providing direct connectivity with the simulator sensory equipment. Measurement data of the flow, pressure, temperature and humidity within the thoracic chamber as well as in the surrounding environment can be monitored during the breathing simulation process. The surrounding environment is of specific relevance if aerosols are applied to the inhaled gas mixture. Signals from the sensors are retrieved by the xPULM Control Software and subsequently transferred to the real-time program for further processing. Generation of impulses controlling motor movement, valve state and vacuum pump performance is based on the recorded values from sensors and set simulation parameters. The breathing pattern, respiratory rate, tidal volume and pressure inside the thoracic chamber can be controlled via the LabVIEW based User Interface. Besides, the flow and pressure characteristics are plotted during the entire simulation runtime. Bidirectional information exchange between both software components provides effective and fast means of breathing simulation control. The simulator was realised with an FPGA-based unit and can, therefore, function as a stand-alone embedded system. The breathing simulation process is controlled without further intervention by the user, which corresponds to the intended general modular and flexible design of the simulator.

The human breathing simulation process is performed under Continuous Rotation Controlled (CRC) mode. For purposes of the CRC control algorithm, any breathing pattern is transformed into a vector of corresponding motor rotation values. Subsequently, motor control impulses matching the desired flow value are generated. Therefore the simulation follows a predefined set of motor rotations without further regulatory inputs from the flow or pressure sensors. For this reason, the volume of the air exchanged during in-/ex-halation phases of a breathing pattern simulation may vary based on the properties of the lung equivalent used. Hence providing the option of investigating the effect of high or low lung compliance and influences of other mechanical properties under steady breathing effort.

## Sinusoidal Respiratory Simulation

Simulation parameters are chosen to represent physiological values, where a breathing rate (*f*) of 12–18 breaths per minute (bpm) is assumed physiological and a tidal volume (*V*_T_) of 500 ml is a typical standard for breathing at rest^30^.

### Parameters of breathing simulations with the mathematical model

The mathematical description as given in chapter 2, models the behaviour of the electro-mechanical simulator during breathing simulations. The mathematical model was implemented in Simulink (MathWorks) environment and supports simulation with adjustable parameters for pressure condition of the surrounding environment, input sinusoidal piston movement profile, leakage flow and balloon-like lung equivalent properties. Following simulation settings of the mathematical model are used for a comparison with the physical version of the xPULM simulator *P*_*a*_ = 1 bar, *V*_0_ = 61 l, *S*_*p*_ = 2.7 dm, *ω*_0_ = 48.3 rad/sec, *C* = 0.2 mol s^−1^ bar^−1^ and dynamic properties of the lung equivalent *ξ* = 35, *C*_*l*_ = 500 dm^3^/bar or 0.5 l/hPa.

### Parameters of the breathing simulations with xPULM

The simulation process is controlled, including breathing parameters settings for each measurement trial, by the xPULM control software. Sinusoidal breathing pattern simulation under CRC mode was tested. Artificial lung equivalents and primed porcine lungs were included in the measurement setup. In the first step, a simplified model of the human lung was realised by two 3 l latex bags. In a second step a higher level of approximation to the more complex structure of human lung was ensured by performing the breathing simulation cycles with primed porcine lungs. Porcine lungs were obtained from a slaughterhouse process. Measurement experiments, each lasting 2 minutes and repeated three times, were performed for all combinations of pulmonary equivalents with different respiratory rates and tidal volumes. The applied set of simulation parameters is summarised in Table 1.

**Table 1:** Overview of the Simulation parameters and their combinations used during the measurements with xPULM.

Simulation Parameters Settings - xPULM	
Breathing Patterns	Sinusoidal in CRC simulation mode
Lung Equivalent	2 × 3 l latex bags, Primed porcine lungs
Frequency	10, 12, 15, 18 bpm
Tidal Volume	400, 450, 500, 550, 600 ml
Initial Pressure	0 hPa (Latex), −6 hPa (Porcine)
Duration	2 min
Repetition	3

The measurements were carried out with an external exhaust air system to realise stable environmental conditions. The following values of the external environment were recorded during the measurements relative air humidity 40–55% RH, temperature 19–21 °C and atmospheric pressure 900–1050 hPa.

Airflow and pressure characteristics during the simulation in CRC mode were recorded for:

- Sinusoidal breathing pattern with two 3 l latex bags in parallel mount.
- Sinusoidal breathing pattern with primed porcine lungs.

### Time normalisation of repetitive cycles

Recorded airflow characteristics of sinusoidal breathing pattern in CRC operation mode were analysed separately for the used lung equivalents, the combinations of breathing frequencies and tidal volumes. Individual respiratory cycles, including in-/ex-halation period, were isolated and time normalised from the raw airflow dataset. Incomplete breathing cycles were discarded from further processing. Time normalisation of repetitive breathing periods includes isolation of the respiratory cycle by finding corresponding intersections of analysed airflow signal with zero isolines. The original time scale is converted to a percentage of a respiratory cycle ranging from 0% to 100%. Time normalisation allows the comparison of repeatable events at any given point in time across all breathing periods. The time normalisation technique is particularly helpful while comparing events that occur with varying frequency or contain different amounts of data samples.

## Evaluation of the Breathing Simulations

**Figure 4:**
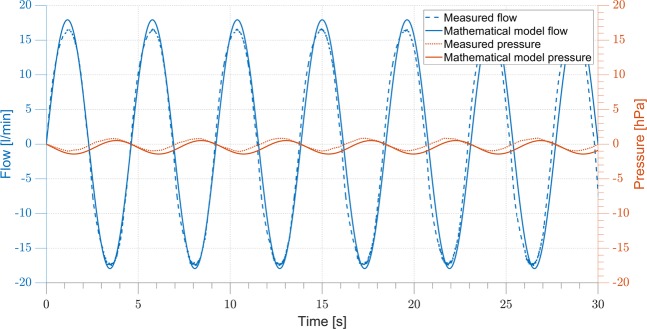
Comparison of the Mathematical model and the xPULM breathing simulation for flow and pressure characteristics for sinusoidal breathing pattern with frequency 13 bpm, tidal volumes 500 ml using two 3 l latex bags in parallel mount as a lung equivalent.

**Figure 5:**
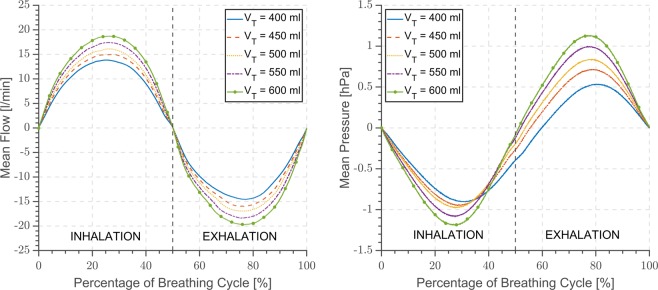
Airflow and pressure characteristics for sinusoidal breathing pattern with frequency 12 bpm, tidal volumes varying from 400–600 ml using two 3 l latex bags in parallel mount as a lung equivalent. The x-axis is normalised in the percentage of breathing cycle where 100% is equivalent to a period of 5 s.

**Figure 6:**
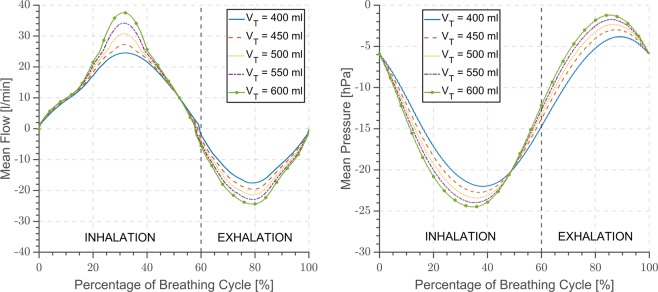
Airflow and pressure characteristics for sinusoidal breathing pattern with frequency 12 bpm, tidal volumes varying from 400–600 ml using primed porcine lungs obtained from a slaughterhouse process. The x-axis is normalised in the percentage of breathing cycle where 100% is equivalent to a period of 5 s.

At first flow and pressure outputs of the mathematical model are compared with the characteristics measured during sinusoidal breathing simulations with xPULM in Fig. 4. Subsequently, additional measurements with xPULM are evaluated to access simulated breathing pattern reproducibility under various conditions. Testing and evaluation of the sinusoidal breathing pattern measured with xPULM includes 20 measurement trials representing changes of five tidal volumes (*V*_T_ = 400–600 ml) at four breathing frequencies (*f* = 12–18 bpm). The results are separated into categories based on the changing breathing simulation parameter and summarised in terms of airflow and pressure characterisation for latex bags in Fig. 5, for porcine lungs in Fig. 6 and for statistical indicators of cycle reproducibility in Table 2. Data with the same simulation parameters have been analysed and evaluated together as one large data set. Breathing cycles have been isolated and evaluated. Mean breathing cycles, as well as standard deviation, have been calculated based on interpolated and time normalised data sets. The standard deviation of |3*σ*| of the isolated breathing cycles has been included to highlight the variation of all breathing signals over time. High reproducibility of flow and pressure characteristics was shown by evaluating breathing cycles (n_Total_ = 3273) with the highest standard deviation |3*σ*| for both, simplified lung equivalents (${{\rm{\mu }}}_{\dot{{\rm{V}}}}$ = 23.98 ± 1.04 l/min, μ_P_ = −0.78 ± 0.63 hPa) and primed porcine lungs (${{\rm{\mu }}}_{\dot{{\rm{V}}}}$ = 18.87 ± 2.49 l/min, μ_P_ = −21.13 ± 1.47 hPa).

**Table 2:** Overview of the evaluated measurements of Sin mode breathing for all tested frequencies and corresponding tidal volumes including number of analysed cycles and statistical indicators for cycle reproducibility.

Sinusoidal breathing pattern in CRC mode; two 3 l latex bags; Primed porcine lungs															
Set breathing frequency [bpm]	Set tidal volume [ml]	Analysed breathing cycles [-]		Mean peak inspiratory flow [l/min]		Mean peak expiratory flow [l/min]		|3*σ*| from mean flow [l/min]		Mean peak inspiratory pressure [hPa]		Mean peak expiratory pressure [hPa]		|3*σ*| from mean pressure [hPa]	
		Latex	Porcine	Latex	Porcine	Latex	Porcine	Latex	Porcine	Latex	Porcine	Latex	Porcine	Latex	Porcine
10	400	60	60	12.24	22.08	−12.56	−13.44	0.69	2.47	−0.78	−22.76	0.43	−4.74	0.63	1.45
	450	60	60	12.95	24.34	−13.72	−16.74	0.53	2.08	−0.83	−23.54	0.50	−3.23	0.20	0.85
	500	60	60	13.95	25.78	−14.92	−18.24	0.75	2.31	−0.86	−23.88	0.62	−2.54	0.19	0.67
	550	60	59	15.20	29.98	−16.06	−20.27	0.76	2.10	−0.90	−24.34	0.73	−1.78	0.20	0.67
	600	59	59	16.24	32.86	−17.01	−21.53	0.71	2.24	−0.96	−24.64	0.81	−1.46	0.19	0.72
12	400	72	72	13.82	24.51	−14.58	−17.56	0.64	2.09	−0.91	−22.01	0.55	−3.73	0.21	0.90
	450	72	72	15.31	27.30	−16.34	−19.63	0.62	2.10	−0.96	−22.76	0.72	−2.91	0.20	0.72
	500	72	71	16.45	30.85	−17.34	−21.42	0.67	2.29	−0.98	−23.44	0.85	−2.27	0.19	0.71
	550	71	71	17.75	34.23	−18.75	−22.94	0.75	2.18	−1.09	−23.99	1.00	−1.69	0.18	0.80
	600	71	70	18.87	37.57	−19.95	−24.39	0.66	2.49	−1.20	−24.53	1.14	−1.15	0.18	0.73
15	400	90	90	16.11	26.66	−17.09	−19.49	0.81	0.87	−0.93	−21.39	0.87	−2.21	0.21	0.59
	450	90	90	17.49	30.53	−18.40	−21.46	0.84	0.92	−1.05	−22.25	0.97	−2.02	0.21	0.64
	500	90	88	18.96	32.74	−19.97	−22.88	0.65	1.32	−1.22	−22.64	1.12	−1.68	0.18	0.67
	550	89	88	20.31	36.88	−21.45	−24.81	0.64	1.06	−1.41	−23.18	1.25	−1.17	0.19	0.74
	600	89	88	21.65	39.28	−22.78	−26.72	0.72	1.61	−1.62	−23.51	1.41	−1.10	0.21	0.74
18	400	108	108	18.21	29.42	−18.84	−21.44	0.72	1.40	−1.14	−21.13	0.99	−3.17	0.21	1.47
	450	108	107	19.81	33.73	−20.62	−23.53	0.80	1.54	−1.39	−22.25	1.12	−2.40	0.20	0.77
	500	107	107	21.40	37.02	−22.14	−25.11	0.76	1.29	−1.61	−22.77	1.29	−1.58	0.20	0.65
	550	107	106	22.76	39.77	−23.51	−28.65	1.01	1.48	−1.87	−23.22	1.43	−0.98	0.19	0.63
	600	107	105	23.98	42.77	−24.61	−32.48	1.04	1.53	−2.16	−23.69	1.53	−0.40	0.21	0.67

### Comparison of the mathematical model and the xPULM breathing simulation

The comparison of the predictions of the established mathematical model and the measured flow and pressure characteristics are both based on the use of a balloon as lung equivalent. In case of the mathematical model a balloon with compliance of 0.5 l/hPa has been used, whereas the included lung equivalent was represented by two 3 l latex bags. The comparison is represented in Fig. 4 and shows a 30 s simulation period with a flow range of ±18 l/min (mathematical model), +16.7 l/min to −17.5 l/min (measured) and a pressure range of +0.5 hPa to −1.4 hPa (mathematical model), +0.8 hPa to −1 hPa (measured). Flow, as well as pressure values, differ in amplitude with −0.5 l/min and 0.3 hPa during exhalation, 1.3 l/min and −0.4 hPa during inhalation respectively. The results show a clear alignment of flow and pressure dependencies for both, mathematical and measured characteristics.

### Breathing simulation with the artificial lung equivalent

Analysed airflow characteristics for sinusoidal breathing pattern simulation using latex bags exhibit standard deviation from the mean airflow waveform of |3*σ*| below 1 l/min for all measured breathing frequencies and tidal volumes. The standard deviation from the mean flow increases slightly with higher breathing frequencies and for greater tidal volumes, reaching the maximum value of 1.04 l/min for *f* = 18 bpm and *V*_T_ = 600 ml. The differences between mean peak inspiratory and expiratory flows increases with growing breathing frequency. The difference of 7.74 l/min for *V*_T_ = 600 ml is highest between breathing frequency of 10 bpm and 18 bpm. A similar trend can be observed in the mean peak expiratory values where the maximal difference is 8.05 l/min for the identical simulation parameters.

Corresponding pressure changes inside the thoracic chamber are characterised by the standard deviation of |3*σ*| below 0.7 hPa at the local maxima for all measured breathing frequencies and tidal volumes. Cycle-to-cycle differences are not dependent on the simulation parameters settings of frequency and tidal volume as their standard deviation does not vary greatly (|3*σ*| < 0.5 hPa). Breathing frequency-dependent increase of the differences between peak inspiratory and peak expiratory pressure corresponds with the results obtained from the airflow sensors. The mean inspiratory pressure difference of −1.20 hPa and the mean expiratory pressure difference of 0.72 hPa respectively was measured between breathing frequency of 10 bpm and 18 bpm for *V*_T_ = 600 ml. Sinusoidal shape of airflow and pressure characteristic is maintained for all measured combinations of tidal volumes and frequencies. Simulation results for volumes varying from 400–600 ml and frequency 12 bpm are depicted in Fig. 5. The in-/ex-halation breathing phase lasts the same time duration resulting in an expected inspiration-to-expiration ration (I: E) of 1: 1. Peak inspiratory flow is reached at 25% and the peak expiratory flow at 75% of the breathing cycle. The pressure inside the thoracic chamber oscillates between ±1.5 hPa during the sinusoidal breathing cycle simulation. The calculated compliance value for the used latex bags is $|{C}_{{\rm{Latex}}}|=\Delta V/\Delta P=0.5\,{\rm{l}}/1\,{\rm{hPa}}=0.5\,{\rm{l}}/{\rm{hPa}}$ at peak inspiratory pressure.

### Breathing simulation with the primed porcine lungs

The standard deviation of |3*σ*| below 6 l/min was recorded while using the primed porcine lungs as a model representing the human lungs. Increase of a difference between individual cycles can be found under the conditions of low breathing frequencies (*f* = 10 bpm and 12 bmp) and small tidal volumes (*V*_T_ = 400 ml and 450 ml). This trend is inverse to the simulation conducted with the latex bags. On the contrary, the difference between mean peak inspiratory and expiratory flow exhibits the same characteristic for both used lung equivalents. The maximal difference for peak inspiratory flow is 9.91 l/min and 10.95 l/min for peak expiratory flow between breathing frequency of 10 bpm and 18 bpm for *V*_T_ = 500 ml, as shown in Table 2.

Pressure changes recorded with the primed porcine lungs have a standard deviation of |3*σ*| under 1.5 hPa for all measurement trials. The expiratory peak pressure is below the atmospheric level, preventing the alveolar regions of the lung from collapsing during the breathing cycle. The shift of the mean peak inspiratory pressure towards negative values compensates the elastic properties of the porcine lungs naturally causing them to stay in a collapsed state. Increase of the simulated tidal volume leads to the decrease of the mean peak inspiratory pressure and concurrently causes a rise of the mean peak expiratory pressure. The complex inner structure, the low compliance due to partly collapsed alveolar regions during exhalation, and the dynamically changing elastic properties of the primed porcine lungs are the reasons for a higher standard deviation of flow and pressure in comparison to the breathing simulations conducted with latex bags. The influence of these parameters is especially noticeable during low volume and frequency simulations when the initial deflation of the tissue originally extracted from the living organism has to be overcome. The state of the extracted porcine lung plays an important role during simulations as any tissue impairment introduces an additional source of leakage. The flow and pressure characteristics are highly reproducible, despite the influence of the tissue properties, and deviate only to a maximum of 2.49 l/min and 1.47 hPa. An overview of the airflow and pressure characteristics during breathing simulation results for both lung equivalents is given in Table 2. However, the simulation with porcine lungs includes many advantages, such as an anatomically accurate approximation of the human lung and its humid inner environment. Moreover, porcine lungs have a comparable lung capacity and are divided into lung lobes. The calculated compliance value for the included primed porcine lung is $|{C}_{{\rm{P}}{\rm{o}}{\rm{r}}{\rm{c}}{\rm{i}}{\rm{n}}{\rm{e}}}|=\Delta V/\Delta P=0.5\,{\rm{l}}/\,-\,23.5\,{\rm{h}}{\rm{P}}{\rm{a}}=0.02\,{\rm{l}}/{\rm{h}}{\rm{P}}{\rm{a}}$ at peak inspiratory pressure.

## Discussion

The xPULM simulator in its current stage includes a bellows system as a driving force for volume displacement, instead of a rigid cylinder piston system as presented by Forjan *et al*.^26^. Due to its real-time data acquisition and processing capabilities this setup redefines the simulation options which were supported to a limited extent by the previous models^27^. These capabilities are enhanced by replacing the computational power of an integrated PC by a stand-alone real-time processing and acquisition unit. Moreover, the newly developed xPULM control software has been tailored to the real-time environment. The boundary conditions of the chosen input signal, in this case, a sinusoidal pattern, are inherently causing the corresponding flow profile of a given frequency and tidal volume. The results presented in this paper, therefore, include flow and pressure measurements over complete sinusoidal breathing cycles with latex bags as shown in Fig. 5 and porcine lungs as shown in Fig. 6. The measurements represent range of *V*_T_ = 400–600 ml and *f* = 10–18 bpm as summarized in Table 2.

The mathematical representation of this electro-mechanical lung simulator has been revised and the simulation parameters adapted to fit the conditions of the measurements. The predicted flow and pressure curves of the mathematical model deviate slightly from the measured values. This can be explained by the fact that the mathematical model represents an optimal situation, meaning ideal conditions. Additionally, leakages within the pneumatic system, including the thoracic chamber and the bellows system, are causing behavioural changes of the introduced lung equivalent. This effect can be seen when inspecting the inhalation phase (Fig. 4), where a higher difference between mathematically modelled and measured peak inspiratory flow can be observed. Additionally, the higher pressure difference, driving the respiratory process, of the mathematical model results in higher peak flows during exhalation.

The latex bags represent a simplified artificial equivalent of human lungs as they contain no inner structure and have a defined volume. Complex tree-like branching of the primary bronchi, typical for the human lungs, is expressed by the main bifurcation separating the latex bags into a left and a right lung. However, such representation assumes both symmetrical bifurcation and lung lobes. In comparison to the human lung, the resistance of a polymer-based bag as lung equivalent is negligible. The resistance of the simplified simulated respiratory tract, consisting of a y-shaped bifurcation and two symmetrical latex bags, would only influence the performed measurements in case of high flows (i.e. flow >120 l/min, airway resistance of 5 hPa/l/s causes a pressure drop of 10 hPa^31^). It is important to note, that the used latex bags are following Laplace’s law for elastic spheres. However, the introduced primed porcine lung represents physiological structures and their characteristics. The behaviour is different as in the asymmetrical setup of the lung, smaller alveoli do not collapse into connected bigger ones, as stated by Prange^32^ and in concordance with physiological lung mechanics. Because of this behaviour, latex bags are introduced into the xPULM system mainly for calibration purposes, rather than for actual lung simulation measurements.

Breathing simulations conducted with the primed porcine lungs are influenced by the properties of the tissue and deviate from the typical sinusoidal curve, as shown in Fig. 6. Airflow during the inhalation period increases gradually until the 20% of the breathing cycle is reached. Previously semi-collapsed alveolar regions expand at this point causing a steep increase of flow and prolonged inspiratory breathing phase. This phenomenon is particularly noticeable during simulations with higher tidal volumes. The expiratory breathing phase is shorter as the airflow expelled from the lung is reinforced by the elastic property of the porcine tissue. The influence of elastic recoil of the primed porcine lung to the breathing simulation is greater in comparison to latex bags. The I:E ration varies with increasing tidal volume reaching approximately the value of 2:1 at *V*_T_ = 600 ml. Such behaviour shows high compliance of the used porcine lung and could be compensated by the inclusion of a closed-loop feedback flow control algorithm. Such an algorithm could continuously adapt motor movement to reach the desired flow and pressure values. Additionally, the inclusion of a fresh porcine lung, with compliance comparable to the physiological range of the human lung, would further enhance sinusoidal respiratory curve approximation.

Focusing on the approach using a polymer bag as lung equivalent, it can be stated that the compliance of the latex bag is influenced by the properties of the material and does not introduce additional forces that would affect the breathing pattern simulations as can be seen in Fig. 5. The calculated compliance value for the used latex bags (${C}_{{\rm{L}}{\rm{a}}{\rm{t}}{\rm{e}}{\rm{x}}}=0.5\,{\rm{l}}/{\rm{h}}{\rm{P}}{\rm{a}}$) is marginally above the physiological range of 0.1–0.4 l/hPa. This high compliance can be explained by the high elasticity of the used polymer material and the absence of any inner structure. In contrast, the included primed porcine lung is characterised by a considerably lower compliance value (${C}_{{\rm{P}}{\rm{o}}{\rm{r}}{\rm{c}}{\rm{i}}{\rm{n}}{\rm{e}}}=0.02\,{\rm{l}}/{\rm{h}}{\rm{P}}{\rm{a}}$), which is caused by the used preservatives influencing the tissue properties and adhering inner structures of the lung tissue. For these reasons, the measurements performed with the latex bags are characterised by high cycle-to-cycle reproducibility with low standard deviation and minor differences across all simulation parameters for both airflow and pressure. The positive expiratory pressure during the simulation was maintained to utilise the entire volume of the latex bags. Due to the missing inner structure of this lung equivalent, its full deflation can be neglected. Fluctuations over time identified during high volume and frequency simulations are caused by the pneumatic system not being completely airtight.

Future challenges include the introduction of vivid porcine tissue, which has not been compromised by the extraction process at the abattoir, or by preservatives. The possible fields of application of the xPULM would increase by the implementation of other, partially also pathological, breathing patterns. The advantages of integrating realistic tissue samples include the possibility of visualising model-machine interactions with ventilation devices to a greater extent. The simulator can then be used for training and testing including the simulation of the patient condition with a physical model where a result of an action is immediately visible, and misuse can result in e.g. tearing of the lung equivalent. The xPULM model can be further applied in simulations for teaching purposes of ventilation where the response and course of action dependent on the change of lung behaviour shall be demonstrated and practised. In medical research, the simulator can be used as an alternative to animal testing. In order to provide an overview of the characteristics of introduced lung equivalents, further measurements will focus on PV loops at low flow rates within the physiological volume range. By these means, different lung conditions can be evaluated and characterised for further breathing simulation. These may include combinations of adult and children lung capacities with or without restrictive and/or obstructive pathologies. The adaptability of the breathing simulation parameters coupled with the use of different lung equivalents represents an important advancement towards anatomically realistic modelling of the respiratory system.

## Conclusion

The xPULM simulator represents a new approach to the respiratory system behaviour modelling, combining the techniques of *in*-*silico*, *ex-vivo* and mechanical models. More specifically the *in*-*silico* part is represented by the mathematical model, the *ex-vivo* component is included by using the primed porcine lung, whereas the mechanical model is the xPULM simulator setup itself. Construction of the simulator has been inspired by the functionality of the human respiratory system under physiological conditions and adapts a key concept of moving the air into and out of the lungs due to the pressure gradient between the atmosphere and the thoracic cavity. Measurements of the simulated sinusoidal breathing patterns demonstrate high reproducibility and stability of the system for all five tested tidal volumes at four breathing frequencies. The respiratory simulations were conducted with simplified lung equivalent and primed porcine lungs. Influence of a lung equivalents’ mechanical properties on flow and pressure characteristic represents processes naturally occurring during the human respiration cycle. This is especially applicable for a primed porcine lung where the extraction process requires increased attention. The results show that the xPULM simulator is capable of reliably capturing the flow and pressure changes for a range of physiological tidal volumes and frequencies with multiple lung equivalents and during sinusoidal breathing simulations.

## Supplementary information

Supplementary video 

## Data Availability

The experimental measurement data and supplementary multimedia material can be provided upon request.
